# The effect of 3D-printed plastic teeth on scores in a tooth morphology course in a Chinese university

**DOI:** 10.1186/s12909-020-02390-0

**Published:** 2020-11-25

**Authors:** Helin Wang, Haokun Xu, Junhua Zhang, Shibin Yu, Meiqing Wang, Jun Qiu, Mian Zhang

**Affiliations:** 1 State Key Laboratory of Military Stomatology, National Clinical Research Center for Oral Diseases, Shaanxi International Joint Research Center for Oral Diseases, Department of Oral Anatomy and Physiology, The Third Affiliated Hospital of the Fourth Military Medical University, 145 Changlexi Road, Xi’an, 710032 Shaanxi China; 2 Department of Medical Education, The Third Affiliated Hospital of the Fourth Military Medical University, 145 Changlexi Road, Xi’an, 710032 Shaanxi China

## Abstract

**Background:**

The tooth morphology course is an important basic dental course. However, it is difficult to fully reflect the three-dimensional (3D) morphological characteristics of tooth structure in two-dimensional pictures in traditional textbooks. The aim of this study was to assess the effect of 3D-printed plastic model teeth in the teaching of tooth morphology.

**Methods:**

Twenty-two undergraduate students who matriculated at the School of Stomatology, the Fourth Medical University, in 2014 and 23 who matriculated in 2016 participated in the study. Each student who matriculated in 2016 was given a full set of fourteen standard 3D-printed plastic model teeth for use during the learning process, and an anonymous questionnaire was used to evaluate the usefulness of the 3D-printed plastic model teeth from the perspective of the students.

**Results:**

There was no significant difference between the two groups in the scores of the theoretical examination or the total score. However, for the score of the sculpted gypsum teeth, the students who used the 3D-printed plastic model teeth in their studies scored significantly higher (*P* = 0.002). More than 90% of the students thought that the 3D-printed plastic model teeth were of great help or were very helpful for mastering the anatomy of teeth and for carving the gypsum teeth.

**Conclusion:**

Standard 3D-printed plastic teeth can effectively assist students in learning tooth morphology by transforming two-dimensional pictures and descriptions in the textbook into a 3D conformation, effectively promoting students’ learning and mastery of tooth morphology and structure. Additionally, the results suggest that 3D-printed plastic model teeth are of great help to the students in mastering and improving their carving skills.

## Background

The tooth morphology course is an important basic stomatology course that mainly covers the composition, classification and anatomical features of a single tooth [1]. To master tooth morphology, students need to actually carve gypsum or wax to deepen their understanding of the characteristic shape of each tooth, establish a 3D concept of teeth, and improve their carving skills for future clinical courses (such as dental prosthetics and cariology/endodontics) and clinical work [2]. Therefore, the teaching of tooth morphology has always been an important but difficult aspect of the teaching of dentistry. A large number of new designs and methods for teaching tooth morphology have been widely used in various stomatological schools [2–5]. However, it is difficult to fully reflect the 3D morphological characteristics of tooth structure in two-dimensional pictures in traditional textbooks.

3D printing is a rapid prototyping technology that is based on digital model files and uses powdery adhesive materials to construct 3D objects through layer-by-layer printing. 3D printing is implemented by a digital printer and is widely used in industrial design, architecture, engineering and construction, automotive design, aerospace engineering and other fields. In dentistry, 3D printing has also been introduced in surgical planning [6, 7] and the production of bone implants [8]. This study compared the application effect of 3D-printing technology in the teaching of tooth morphology by using 3D-printed plastic model teeth that matched the traditional tooth morphology textbooks and conducted a questionnaire survey of the students’ feelings about the use of the 3D-printed plastic model teeth.

## Methods

The study was approved by the Institutional Review Board (IRB) at the Fourth Military Medical University ZL201425.

### Tooth morphology teaching process

At the Fourth Military Medical University, dentistry professional undergraduates take courses on tooth morphology in their second year. The “Dental Gypsum Sculpture Training Course”, published by People’s Medical Publishing House in 2008, is used as teaching material. The courses are composed of 70 credit hours, including seven credit hours of theory on a pandect of tooth morphology and the anatomical features of incisors, canines, premolars and molars. During the 61 credit hours of gypsum dental carving practice, central incisors, lateral incisors, canines, first premolars, second premolars, first molars and second molars of the right maxilla and mandible are carved, with 3–6 credit hours allotted for each tooth. The theoretical examination is worth two credit hours.

In the online classroom at our school, the chapter on tooth morphology in the online course “Oral Anatomy and Physiology”, which was developed by our teaching and research office, is used for instruction. In the class, the teacher first explains the main morphological characteristics of each tooth to the students in approximately 1 h by using a combination of multi-media materials available in the online course. Then, the students work independently to learn according to their own mastery of the relevant knowledge. They can choose their own learning methods from text descriptions, dental pictures, videos, dental animations and other materials. During this process, the teachers are responsible for answering questions. Before the end of the class, the teacher takes 10 to 15 min to summarize the main points of the morphology of each tooth.

In the class on gypsum dental carving practice, the teacher first spends approximately 5 min reviewing the main morphological characteristics and details that need attention in carving. Another 30 min are used for instruction on gypsum tooth carving or watching videos of tooth carving. Then, the students carve a tooth according to the teacher’s instructions and the step carving methods in the textbook while the teacher moves around the classroom and answers questions.

### Tooth morphology examination process

Theoretical assessment is carried out by means of a closed-book written examination. The main assessment content is the anatomical morphological characteristics of each tooth and the morphological comparison between different teeth. The question types vary, with 30% being multiple choice questions, 20% being noun interpretation (explanation or description of a given word or glossary term), and 50% being short-answer questions. The full score of the theoretical assessment is 100, accounting for 50% of the total score of the course.

After the theoretical assessment, students are required to hand in a set of sculpted gypsum teeth. The score is based on the accuracy of the shape and the precision of the detail carving in the gypsum teeth. The score of each tooth is as follows: 6 points for each maxillary central incisor/lateral incisor/canine and mandibular central incisor/lateral incisor/cusp (shape: 4 points, details: 2 points); 7 points for each maxillary first premolar/second premolar and mandibular first premolar/second premolar (shape: 5 points, details: 2 points); and 9 points for each maxillary first/second molar and mandibular first/second molar (shape: 6 points, details: 3 points).

### Research object and method

Twenty-two undergraduate students who matriculated in 2014 and 23 students who matriculated in 2016 at the School of Stomatology, the Fourth Medical University, participated in the study. All students were engaged with the above teaching and assessment methods.

The two groups of students had the same teachers in the theoretical course and the same teachers in the practical course on gypsum tooth carving (the teachers were recording videos of gypsum tooth carving for a national textbook titled “Dental Gypsum Sculpture Training Course”). The theoretical examination questions were set by the same teacher for both groups (the proportion of the different types of questions was the same, and the overall difficulty was basically the same). The gypsum tooth carvings were assessed by two teachers independently. The average score of each tooth with a score difference of less than 2 points was taken as the score of that tooth. If the score difference was more than 2 points, a third teacher independently scored the tooth again, and the average of the two scores with the most similar values was taken as the score of the tooth.

Before the beginning of this course, each student who matriculated in 2016 was given a set of 3D-printed plastic model teeth. At the end of the course, the students were given an anonymous questionnaire to investigate the usefulness of the 3D-printed plastic model teeth in the course.

### Preparation of the 3D-printed plastic model teeth

Before the course, the teacher carved a full set of 14 standard gypsum teeth as models and used the models in a model scanner (3shape) to obtain the data for the tooth printing. 3D printers (Shaanxi hengtong R200) using PLA plastic were used to print the plastic model teeth according to the data information from the model.

### Statistics

SPSS Software for Windows version 24.0 was used for statistical analysis of the data. The scores of the two groups of students in the theoretical assessment and gypsum tooth carving and the total scores were in line with the normal distribution and homogeneity of variance. The scores of the two groups of students in the theoretical assessment and gypsum tooth carving as well as the total scores were tested by a t test, and *P* < 0.05 was considered statistically significant.

## Results

### Comparison of scores between the two groups of students

Between the two groups of students, there was no significant difference in the theoretical examination scores and the total score. However, for the scores of the sculpted gypsum teeth, the students who matriculated in 2016 who used the 3D-printed plastic model teeth in their class scored significantly higher than those who did not use the model teeth. (Table 1).

**Table 1:** Comparison of scores of tooth morphology between the two groups of students

Class	Theoretical examination score	Gypsum tooth-carving score	Total score
Students matriculated in 2014 (22)	87.28 ± 4.85	83.18 ± 4.51	85.23 ± 3.46
Students matriculated in 2016 (23)	86.26 ± 6.01 (*P* = 0.575)	87.57 ± 4.55 (*P* = 0.002)	86.91 ± 3.97 (*P* = 0.127)

### Survey results of students who matriculated in 2016

In this survey, only 13.04% of the students thought that the traditional two-dimensional pictures in the textbook clearly expressed dental morphological features. Only 39.13% of the students could identify the correct locations and names of the anatomical structures in the two-dimensional picture, and 65.22% of the students could not completely match the pictures with the corresponding text description in the textbook. These findings suggest that there are still many defects and deficiencies in traditional textbooks regarding 3D structures, such as the anatomy of tooth morphology.

A total of 91.37% of the students thought that the use of the 3D teaching aids and video animation in tooth morphology was more helpful; 95.65 and 91.37% of the students thought that the 3D-printed plastic model teeth were of great help or were very helpful, respectively, for mastering the anatomy of teeth and for carving the gypsum teeth. The students believed that the main advantage of the 3D-printed plastic model teeth was that the anatomical morphology and characteristics of the teeth were clear. Compared with the two-dimensional pictures in the textbook, the 3D-printed plastic model teeth could be used as direct examples when carving, which facilitated the direct comparison between different teeth. (Table 2).

**Table 2:** Questionnaire survey results of students who matriculated in 2016

Question	Options			
1. Do you think the pictures in the textbook clearly express the morphological characteristics of the teeth?	Absolutely 0%	Mostly 13.04%	Somewhat 43.48%	Not at all 43.48%
2. Are you fully aware of the names and locations of the anatomical structures in the pictures?	Absolutely 0%	Mostly 39.13%	Somewhat 56.52%	Not at all 4.35%
3. Can you find the text that corresponds to the pictures?	All 0%	Mostly 34.78%	Somewhat 65.22%	Not at all 0%
4. Do you think the teaching aids and video animation are helpful in understanding the anatomy of teeth?	Very helpful 60.87%	Mostly helpful 30.34%	Less helpful 8.69%	Not helpful 0%
5. Do you think the 3D-printed tooth models are helpful in mastering the anatomy of teeth in class?	Very helpful 78.26%	Mostly helpful 17.39%	Less helpful 4.35%	Not helpful 0%
6. Do you think the 3D-printed tooth models are helpful for carving the plaster teeth?	Very helpful 86.96%	Mostly helpful 4.35%	Less helpful 8.69%	Not helpful 0%
7. What do you think are the advantages of a 3D-printed dental model? (multiple choice)	It is more stereoscopic than pictures in textbook 91.30%	The anatomical morphology and features are clear 73.91%	It could be used as a direct sample when carving 100%	It facilitated direct comparison between different teeth 86.96%

## Discussion

Dentistry is a subject with strong practical components, and the cultivation of students’ practical abilities is incorporated into the whole teaching process [9, 10]. According to a survey, 98.63% of dental students in China had not been exposed to relevant knowledge of tooth morphology before their sophomore year, and 82.19% of dental students had not received hands-on training on the same subject [11]. In contrast, in the United States, Japan, Europe and other developed countries, dental courses such as clinical probation and tooth carving are arranged towards the beginning of the curriculum, soon after entrance into the school, so that students can have access to dental knowledge and relevant skills as early as possible [12–16].

In 2001, our university was the first in China to start offering “tooth morphology” as an extension course for second-year dental students. This offering mainly improved the students’ practical ability through the carving of gypsum teeth, and the course simultaneously gave students a basic grasp of the anatomy of teeth, thus laying a solid foundation for later relevant professional courses and clinical work [17]. Previous teaching experience suggests that by the time a student completes 70 credit hours on tooth morphology, the student’s manipulative ability will have greatly improved, and he or she will have a degree of mastery over the anatomical features of the teeth. However, when limited by traditional teaching materials, including the limitations of two-dimensional pictures, students find it difficult to form 3D tooth structural configurations in their minds. A previous questionnaire also indicated the following major problems: (1) Students could not mentally convert the two-dimensional pictures of teeth in the textbook into 3D tooth forms. When carving gypsum teeth, they could not score well because their overall understanding of the teeth was poor. (2) Depending on the teacher and the availability of physical samples after class when practising carving, it was difficult to complete the tooth carving independently. (3) Due to the poor quality of the printed pictures in the textbook and the interference of light and shadow in the pictures, the dental structural details in the pictures were insufficient, and it was difficult for students to master the morphological characteristics of teeth when carving.

Aware of the above problems, our teaching and research section has been carrying out teaching reform in tooth morphology for a long time, including introducing 3D animated demonstrations of teeth into the learning process, increasing the number of contact hours during the carving of the details of gypsum sculptures and giving students highly precise images of teeth as examples [11, 17]. However, it is still difficult to solve the abovementioned three major problems. The emergence of 3D-printing technology has provided new methods for solving these problems in the learning process. After the model scanner and 3D printer were installed in our teaching and research office, we began to explore the possibility of 3D printing in teaching tooth morphology. Our teaching and research section is tasked with compiling a unit for the national textbook titled “Dental Gypsum Sculpture Training Course”. The pictures in the textbook and the videos of the tooth-carving process were created by senior faculty in the teaching and research section who had long-term experience in teaching tooth morphology. Using 3D-printing technology to print completely standard teeth in accordance with the pictures and videos used in national textbooks is of mutual advantage for our teaching and research sections.

After applying the standard 3D-printed plastic model teeth in the daily instruction on tooth morphology for students who matriculated in 2016, we found the following: (1) When taught the theoretical and morphological characteristics of teeth, the students all consciously held the standard 3D-printed plastic model tooth in their hands and compared it with the pictures, textbook and teaching materials. (2) In the practical training in gypsum tooth carving, the students all used the standard 3D-printed plastic model teeth as carving references and regularly compared the gypsum teeth they carved with the 3D-printed plastic model teeth during each step of the carving process to adjust their carving appropriately. Thus, the 3D-printed plastic model teeth can effectively assist students in learning tooth morphology by transforming the two-dimensional pictures and descriptions in the textbook into 3D conformations, effectively promoting students’ learning and mastery of tooth morphology and structure. Compared with the scores of the students who matriculated in 2014, the average dental gypsum sculpture score improved significantly (by 4.39 points) for the students who matriculated in 2016 after the 3D-printed plastic model teeth were added to the learning process. In particular, the quality of the carved teeth details improved significantly, with a more suitable ratio of dental crown and root, more accurate position of cusps and ridges, more precise area and depth of fossa, smoother dental cervical line and clearer grooves and pits in occlusal surfaces. The study showed that the 3D-printed plastic model teeth were also of great help for the students in mastering and improving their carving skills. In addition, the 3D-printed plastic model teeth could effectively overcome the past weaknesses of tooth morphology instruction. The students were able to use the intuitive 3D structure and clear details of the teeth as a reference for equal proportions when carving. It was also easy to directly compare the characteristics of different teeth, which was widely accepted and welcomed by the students.

It has been less than 20 years since tooth morphology was established as an independent course in China. Only a few colleges and universities have set up this course independently, and most of them adopt traditional teaching methods. Until now, no paper in English on the teaching and research of tooth morphology in China has been published in journals, but there are many foreign papers on the teaching and research of tooth morphology that provide good comparisons. Nance et al. [18] assessed the use of an instructional DVD on tooth carving compared to traditional laboratory instruction. The students positively assessed the use of the DVD as a teaching resource, but their carving ability did not improve. Kwon et al. [19] compared the feedback of two digital evaluation systems for assessing the carving projects of students with traditional teaching technologies and found no improvement in the carving performance of students. We used only video, pictures and other visual materials in the early teaching, similar to other studies [4, 20, 21]. However, due to the lack of a basic grasp of tooth morphology, it is difficult for beginner students to apply these materials to improve their tooth-carving skills. A technique in which the tooth was carved step-by-step alongside the professor was found to improve the quality of students’ carving in another study [22]. Therefore, in the later teaching, we mainly added a carving demonstration by the teacher and gave the students step-by-step instruction and guidance in tooth carving. However, due to the class period and the number of teachers, the students’ carving ability was only slightly improved. The 3D-printed plastic model teeth can make up for the above deficiencies. They can be used as an effective reference and at the same time allow students to practice carving freely in their spare time to improve their carving ability. In addition to acting as a teaching method, this approach improved the students’ self-directed learning and self-assessment. Previous studies have claimed that the main advantages of digital resources include unrestricted access to information regardless of place and time, thus allowing flexible study time [23, 24].

Interestingly, the average score of dental gypsum sculpture improved significantly (by 4.39 points) for students who matriculated in 2016, but the mean theoretical assessment score was no different than that for students who matriculated in 2014. Similar to our results, Alzahrani et al. [25] revealed that dental carving scores were higher for students of the experimental group taught by new methods, but the mean theoretical knowledge scores did not improve significantly compared with those for the traditional lecture group. It might be that in contrast to the test of carving skill, the test of theoretical knowledge examines students’ memorized information about tooth morphology, which is not based on carving ability.

In addition, some other teaching methods that do not focus on carving skill have also promoted mastery of dental carving. Chutinan et al. [26] implemented a flipped classroom teaching model involving a self-study period before each class, in-class activities, and discussion sessions and compared it to the previous existing model. The carving score of the traditional lecture group was significantly lower than that of the flipped classroom group. Obrez et al. [27] compared a revised dental anatomy module focusing on a review of content before class, small-group discussions of clinically relevant dental anatomy topics, and more clinically realistic exercises in the laboratory with the module before the revision (control group). The carving examination generated significantly higher scores in the experimental group than in the control group. In the above two studies, a variety of educational resources were explored in student-driven approaches. The authors used books, atlases, manuals, CD-ROMs, and video lectures as pre-class resources to produce significant improvement in students’ dental carving ability. The above data suggest that the combination of a flipped classroom and other teaching methods with standard 3D-printed teeth may further improve students’ mastery and carving skills, which is worth further experimental research in the future.

In the process of teaching tooth morphology at other colleges and universities in China, commercially produced model teeth have been used as a means of teaching exploration [28, 29]. However, the commercial model teeth have several major problems: (1) The size of the model teeth does not match the size of the gypsum blocks the students use in their carving. The commercial models are too large or too small, and it is therefore difficult to use them as an equal-scale reference when carving. (2) The production data used to create the commercial model teeth are derived mainly from tooth morphology measurements in foreign countries, which differ from the average data of adult tooth measurements in China that are used in the national textbooks, resulting in morphological differences between the commercial model teeth and the picture examples in the textbooks. (3) The price of the commercial model teeth is relatively high, usually more than ¥300 (approximately $45 or €38), which creates an economic burden for both the students and the teaching management departments. The above three problems can all be solved with our 3D-printed plastic model teeth.

We have carried out only a preliminary exploration of 3D-printed plastic model teeth in the teaching of tooth morphology. The number of students using 3D-printed plastic model teeth is still lower than the number of those using more traditional methods. The current teaching methods and content need systematic improvement and are not yet completely compatible with the characteristics of the 3D-printed plastic model teeth used in the tooth morphology course. Teachers need additional experience with using 3D-printed plastic model teeth in their teaching process. In the future, it will be necessary to develop more explicit requirements for the use of 3D-printed plastic model teeth in the teaching of tooth morphology, and the teaching level and quality must be substantially improved through continuous improvements in practice.

## Conclusion

In this study, the score of sculpted gypsum teeth was higher for the students who used 3D-printed plastic model teeth. More than 90% of the students thought that the 3D-printed plastic model teeth were of great help or were very helpful in mastering the anatomy of teeth and in carving the gypsum teeth. 3D-printed plastic model teeth can effectively assist students in learning tooth morphology by transforming the two-dimensional pictures and descriptions in the textbook into 3D conformations, effectively promoting students’ learning and mastery of tooth morphology and structure as well as being of great help to the students in mastering and improving their carving skills.

## Data Availability

All data will become available if requested.
